# The complete mitochondrial genome of *Cyclina sinensis* (Veneroida:Veneridae)

**DOI:** 10.1080/23802359.2016.1149786

**Published:** 2016-03-28

**Authors:** Pengzhi Dong, Guangyin Ma, Lianying Chang, Yan Zhu, Xiaoxuan Tian

**Affiliations:** a Tianjin State Key Laboratory of Modern Chinese Medicine, Tianjin University of Traditional Chinese Medicine, Tianjin, China;; b The First Teaching Hospital of Tianjin University of Traditional Chinese Medicine, Tianjin, China;; c Molecular Cardiology Research Institute, Tufts Medical Center, Boston, MA, USA

**Keywords:** *Cyclina sinensis*, mitochondrial genome, Veneridae

## Abstract

*Cyclina sinensis* belongs to the family Veneroida, Veneridae, a marine mollusks having high economic value. It is distributed in the coastal waters of Southeast Asia. In recent years, the recourses of *C. sinensis* have been severely depleted due to overfishing and pollution. Genetic divergency has also been reported. It is necessary to protect this bivalve from the hazards. In this study, the complete mitochondrial genome of *C. sinensis* has been determined. The total number of nucleic acids were 21 799 bp. The genome contains 13 protein-coding genes, 22 tRNA genes, 2 rRNA genes and a non-coding control region. The phylogenetic analysis was conducted with 11 related species and confirmed the classification status.

*Cyclina sinensis* belongs to family Veneridae. It is an important economic marine mollusk distributed in North Korea, Japan, Ryukyu Islands, Southeast Asia and the north and south coast of China. *Cyclina sinensis* have been considered as rich source of protein for Chinese people living near the sea. Besides, it is also considered as an important ingredient in Chinese medicine, whose shell was used to protect the wounds and had anti-inflammatory effect. Thus, it is necessary to protect this species from the hazards caused by the human activities. Besides that, genetic divergence was also observed (Yoon 2012). In order to preserve this economic bivalve, genetic tools should be employed. Recently, complete mitochondrial genome has been considered a useful tool for population genetic and phylogenetic studies (Cameron 2014).

In this work, the complete mitochondrial genome of *C. sinensis* was determined using high-throughput sequencing methods (GenBank accession number: KU097333). Individual of *C. sinensis* were purchased from Dadonghai Seafood (voucher ID: QG-1). The complete genome is 21 799 bp in length. The composition of the whole genome is 26.2% A, 46.8% T, 7.8% C and 19.1% G. Content of AT is 73.1% and GC is 26.9%. It contains 13 protein-coding genes (PCGs), 22 tRNA genes and 2 rRNA genes. In the family of Veneridae, mitochondrial genome of 11 species have been sequenced up to date. Interestingly, only 5 mtDNA including *C. sinensis* have 13 PCGs, others have 12 PCGs lacking *ATP8* gene. The longest gene of *C. sinensis* mitochondrial genome is *ND5* containing 1 731 bp and the shortest is *ATP8* gene which is 117 bp long. Twelve PCGs start from ATG while CytB start from ATA. *ND5*, *COX1*, *ND2* and *COX3* use TAG as a stop codon. *COX2*, *CytB*, *ND1*, *ATP6*, *ND6*, *ND3*, *ND4L* and *ATP8* use TAA as a stop codon. Besides, only *ND4* uses incomplete stop codon, TA–. Typical tRNA cloverleaf secondary structure is predicted by MITOS Web Server. The largest non-coding region is putative control region, which is 3 225 bp in length. Total gaps of 2 064 bp were observed in the genome. As shown in Figure 1, the phylogenetic analysis of Veneridae was conducted (Price et al. 2010).

**Figure 1.**
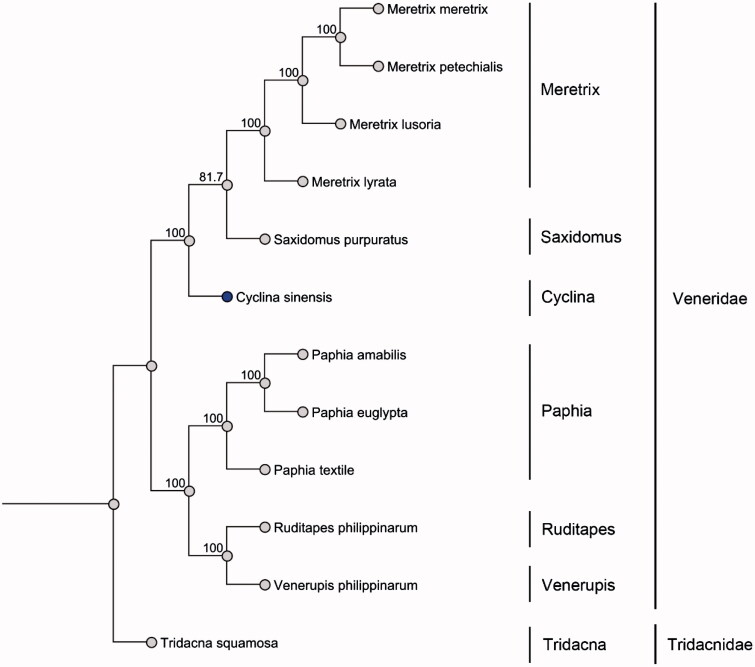
Twelve mitochondrial DNA from Genbank were used to build the maximum-likelihood phylogenetic tree based on sequences of translated mitochondrial proteins. One-thousand replicates of bootstrap were set. MUSCLE was used to align the sequences. Sequence data used in the study are the following: Meretrix lusoria, NC_014809; Meretrix lyrata, NC_022924; Meretrix meretrix, NC_013188; Meretrix petechialis, NC_012767; Paphia amabilis, NC_016889; Paphia euglypta, NC_014579; Paphia textile, NC_016890; Ruditapes philippinarum, KT001084; Saxidomus purpuratus, NC_026728; Tridacna squamosal, NC_026558; Venerupis philippinarum, NC_003354.
